# Representation of Multiplication Facts-Evidence for partial verbal coding

**DOI:** 10.1186/1744-9081-7-25

**Published:** 2011-07-08

**Authors:** Korbinian Moeller, Elise Klein, Martin H Fischer, Hans-Christoph Nuerk, Klaus Willmes

**Affiliations:** 1 Knowledge Media Research Center (IWM-KMRC), Tuebingen, Germany; 2 Institute of Psychology, Eberhard Karls University, Tuebingen, Germany; 3 Department of Neurology, Section Neuropsychology, RWTH Aachen University, Aachen, Germany; 4 School of Psychology, University of Dundee, Dundee, UK

## Abstract

**Background:**

The current view in numerical cognition research is that multiplication facts are stored and retrieved in a phonological code. Consistent with this view, it was found that multiplication could be impaired by a phonological but not by a visuo-spatial loading task. However, because the authors used an active production task, it remained unclear whether concurrent articulation impaired either access to multiplication facts or their retrieval.

**Methods:**

In the current study, we investigated the influence of concurrent articulation on multiplication fact knowledge without active production of multiplication results.

**Results:**

In a number bisection task, number triplets, which are part of a multiplication table, were classified faster as being correctly bisected than other triplets. Interestingly, concurrent articulation led to a relative slowing of the multiplicative triplets which reduced the multiplicativity effect.

**Conclusions:**

This result indicates that concurrent articulation modulates access to phonologically stored multiplication facts and corroborates the notion of multiplication facts being represented in an at least partially verbal code.

## Introduction

According to the most influential model of number processing, the Triple Code Model by Dehaene and colleagues [1-4], numerical cognition rests on the representation of numerical magnitude and its further processing by arithmetic procedures, as well as on arithmetical facts stored in long-term memory. The magnitude of any number is assumed to be represented in an analogue magnitude code along a left-to-right oriented mental number line. In order to solve subtraction or multi-digit addition problems, the respective magnitudes of the operands have to be manipulated [2]. Furthermore, arithmetic facts are presumably represented as automatically accessible verbal associations of rote-memorized long-term memory entries. Thus, multiplication with small numbers may even be carried out without explicitly activating the magnitude code of the numbers; instead, the correct result may be accessed directly and retrieved from memory [2,3,5]. The latter account has been described as the phonological storage hypothesis of multiplication facts. In the strongest version of this account, multiplication facts are assumed to be represented phonologically, but a less stringent version suggests that multiplication facts are represented as verbal associations of filled word frame representations of operands without explicit phonological coding (comparable to the lemma level in Levelt's language production model [6]; cf. Dehaene & Cohen [2,3,5]^1^. To date, most evidence corroborating either version of the phonological storage hypothesis comes from single-case studies evaluating arithmetic performance of brain-damaged patients (e.g., [2,3,5]; but see [7,8] for inconsistent patient data). However, there is now first longitudinal developmental evidence also suggesting phonological coding of arithmetic facts [9]. Nevertheless, only a few experimental studies addressed the nature of the mental representation of multiplication fact knowledge. One prominent exception is a study by Lee and Kang [10], which will be introduced briefly in the following (see also [11]).

Lee and Kang [10] observed an intriguing dissociation in a dual task paradigm where participants had to repetitively utter a non-word string during mental arithmetic: While the processing of subtraction problems was not affected by concurrent articulation, multiplication performance was reliably impaired. The authors interpreted their finding to indicate that an "auditory-verbal code may be used for multiplication, which was therefore differentially suppressed by a phonological dual task" ([10], p. B67). However, the authors themselves did not specify at which processing stage this interference may have occurred. Because participants had to actively produce the arithmetic result it cannot be decided whether concurrent articulation indeed impaired access to/retrieval from long term memory, or rather the activation of stored multiplication facts in long-term memory. On the one hand, it may be the case that multiplication facts are stored in an auditory-verbal fashion (as opposed to non-verbal coding). On the other hand, non-verbally stored multiplication facts may just be recoded verbally during the access/retrieval process. Both of these hypotheses may account for the impairment observed by Lee and Kang [10], but they cannot be further differentiated because of the experimental setup used (see e.g., [12], for the differentiation of storage of vs. access to mental representations in cognitive neuropsychology).

The present study set out to further narrow down the processing stage at which the impairment of multiplication under concurrent articulation may have occurred. We employed a verification version of the number bisection task (NBT) that differs from the task used by Lee and Kang [10] in two important aspects: First, this version of the NBT did not require active production of any multiplication result. Second, because the default strategy to solve the NBT relies on magnitude manipulation [5], multiplication fact knowledge is actually task-irrelevant. Therefore, no intentional access to multiplication facts should occur and the verbal output lexicon should not be activated. Nevertheless, Nuerk, Geppert, van Herten, and Willmes [13] observed a reliable influence of multiplication fact knowledge on performance in the NBT (see also [14-16]). Classifying a number triplet as being arithmetically correctly bisected by its central number (e.g., 21_26_31 vs. 21_26_29) was reliably faster when the triplet was part of a multiplication table as compared to when it was not (e.g., 18_21_24 vs. 19_22_25). Nuerk and co-workers [13] termed this finding the multiplicativity effect because it was assumed to originate from the additional recruitment of multiplication fact representations. These multiplication facts are supposed to be stored in an auditory-verbal format and to corroborate NBT task performance which was thought to primarily rely on magnitude representations. Thus, the observation of the multiplicativity effect suggested an interactive recruitment of numerical representations when performing the NBT, rather than a strictly one-task one-representation account (see also [14,16]).

### Representational characteristics of the multiplicativity effect

It is important to note that the multiplicativity effect cannot be explained by a simple counting strategy (i.e., count by 2, 3, 4, etc.) because such a strategy would be applicable to non-multiplicative triplets such as 19_21_23, 20_23_26, or 19_23_27 as well. However, for such non-multiplicative triplets no facilitation was observed, which in fact reflects the multiplicativity effect. Three further sets of empirical evidence from studies employing exactly the same task indicate that it is indeed the retrieval of multiplication fact knowledge that drives the multiplicativity effect. First and most importantly, Wood and colleagues [14] reported that the processing of multiplicative triplets specifically modulated the fMRI signal within the left angular gyrus. As the left angular gyrus is generally considered to be involved in the processing of multiplication facts (e.g., [4,17-19]), this observation strongly suggests that the multiplicativity effect originates from the recruitment of multiplication fact knowledge. This interpretation is further corroborated by the fact that for non-multiplicative triplets increased activation within the bilateral intraparietal sulci was found-an activation pattern usually attributed to increased demands on magnitude processing. Second, based on the eye fixation pattern recorded while participants performed the NBT, Moeller and colleagues [15] were able to associate the recognition of multiplicativity with fast and automatic activation of multiplication fact knowledge, because it specifically influenced eye-movement measures sensitive to stimulus driven bottom-up processing. It is important to note that such an interpretation is in line with recent data by Rusconi and co-workers (e.g., [20-22]) who observed multiplication products to be primed by their operands. Finally, Moeller and co-workers [16] further substantiated this finding using the higher temporal resolution of EEG methodology. The authors observed that from about 250 ms post-stimulus on upper alpha power was significantly reduced at left parietal electrodes but not at right parietal electrodes. Generally, upper alpha desynchronization is associated with the retrieval of information from long-term memory (including number magnitude information, e.g., [23]). Following this rationale, the authors interpreted their results as suggesting that the process of coming to the correct solution differs between the left and the right hemisphere. While the strong activation of right parietal sites indicates that in the left hemisphere magnitude related processes are elicited to derive the correct response, reduced activation in left parietal areas suggests that such demanding magnitude manipulations are bypassed by the retrieval of multiplication facts. Considering this converging empirical evidence, it seems reasonable to assume the multiplicativity effect to originate from the recruitment of multiplication fact knowledge in the NBT^2^.

The absence of explicit verbal recoding of multiplication facts in the NBT allows for a further specification of Lee and Kang's [10] dissociation between arithmetic operations (i.e., multiplication vs. subtraction) and the kind of loading task used (i.e., verbal vs. visuo-spatial). This specification pertains to the neuropsychological distinction between the mental storage of knowledge vs. access to this knowledge ([12,24,25]; see [26,27] for recent applications in numerical cognition); and thus, the question whether any performance differences observed may be due to impaired access to multiplication facts (i.e., hampered retrieval or production) and/or an impairment of the underlying storage of multiplication fact knowledge (i.e., the representation itself). For the case of arithmetic, Benson and Denckla [28] described a patient with an astrocytoma in the left posterior parietal cortex. On the one hand, the patient's calculation performance was specifically impaired for multiplication. On the other hand, the impairment was only observed as long as the answer to the problem had to be produced either orally or in written form: when asked to choose the correct answer from a list of solution probes (i.e., a multiple choice paradigm) the patient performed much better. For instance, "with the written problem 4 + 5 he said eight, wrote 5, and chose 9 from the multiple choice list" ([28], p. 98). This data pattern suggests a dissociation between stored representation of multiplication facts and access to or retrieval of these facts (see also Girelli [29]). Interestingly, the case report by Benson and Denckla [28] already provided first evidence for a specific involvement of auditory-verbal codes in multiplication. Wrong verbal repetition/recoding of the presented problems were particularly detrimental for multiplication problems. Often, the patient stuck to the verbally recoded problem and tried to produce or actually produced the answer to the verbally recoded problem (see also Case 1 of Benson & Denckla [28]). This prominent role of verbal codes for multiplication fact knowledge was further supported by more recent patient studies (e.g., [3,5]) but only few experimental studies in healthy participants (e.g., [10]).

#### The present study

In the current study we aimed at evaluating the nature of the representation of multiplication fact knowledge and in particular the auditory-verbal component of multiplication facts. Therefore, we introduced an articulatory suppression condition to the NBT to evaluate the effect of concurrent articulation on the multiplicativity effect. Generally, as regards effects of articulatory suppression/auditory distraction, it has to be noted that not only slowing effects on overall RT (as observed by Lee & Kang [10]) have been reported. For instance, already Cassel and Dallenbach [30] summarized that "the effect of 'distraction' [...] may inhibit, and lengthen the reaction; it may facilitate, and shorten the reaction; or it may [...] have no effect at all." (p. 143). In particular for rhythmical, regularly timed distractors such as a metronome even speeding instead of slowing was observed ([31,32] for speeding in numerical tasks due to concurrent articulation). However, it should be considered that we were not interested in the effect of concurrent articulation on overall performance. Instead, we were particularly interested in the specific effect of articulatory suppression on the multiplicativity effect. Importantly, Saito [[33], see also [34]] argues that it cannot be assumed that concurrent articulation does suppress and/or inhibit the processing of auditory-verbal material in the phonological loop entirely, but nevertheless impedes processing in the phonological loop severely. Therefore, articulatory suppression cannot be assumed to eliminate the multiplicativity effect completely. Instead, in line with the argument of Saito [33] that concurrent articulatory operations may not be "sufficient to abolish the phonological loop completely, they are enough to decrease the recall to a certain level" (p. 575), modulation of performance by concurrent articulation will be considered to indicate that task performance is at least partially mediated by verbally coded representations (see also [10]).

Because of the only implicit recruitment of multiplication fact knowledge in the NBT (i.e., no activation of the retrieval-related output lexicon) the following hypotheses were put forward: First, a reliable interaction of articulatory suppression and multiplicativity with the multiplicativity effect being reduced or eliminated by articulatory suppression would indicate that multiplication fact knowledge is at least partially represented in a verbal code, thus corroborating the phonological storage hypothesis. Second, when multiplication facts are represented in a nonverbal code (e.g., magnitude related or visuo-spatial) and when only access to multiplication fact knowledge involves auditory-verbal recoding, then we should observe no interaction of concurrent articulation and multiplicativity in the NBT at all. In other words, because the verbal output lexicon is not activated in the NBT when multiplication facts are accessed in a semantic or visuo-spatial manner, no interference between articulatory suppression and the retrieval process should occur.

In summary, we hypothesized that when multiplicativity interacts with concurrent articulation (being either reduced or eliminated) this would indicate at least partial verbal storage of multiplication facts in long-term memory.

## Method

The study was conducted in accordance with the ethical guidelines of the British Psychological Society (BPS).

### Participants

12 right-handed students (six females, six males) of the University of Dundee, Scotland/UK, were paid for their participation in the experiment (mean age: 21.3 years; SD: 2.4 years). All participants reported normal or corrected-to-normal vision.

### Tasks

The primary task in the current study was the Number Bisection Task (NBT) in its verification version, as introduced by Nuerk et al. [13]. The NBT requires participants to determine whether the central number of a triplet also represents the arithmetic mean between the two outer numbers or not (e.g., 21_24_27 vs. 21_25_27). As a secondary task participants performed a concurrent articulatory loading task by repeating the non-sense syllable string "pataka" in a staccato fashion at a rate of approximately three syllables per second. This particular string was chosen due to its distinct phonemes, each of which requires the participants to adopt a series of different articulatory postures, thus ensuring the effectiveness of articulatory suppression [33].

### Stimuli and design

The experimental design was a 2 × 2 design for correctly bisected triplets involving the factor multiplicativity (i.e., e.g. 12_16_20 vs. 15_19_23) with bisection range (i.e., the numerical distance between the outer numbers of the triplets held constant across multiplicatively related and unrelated triplets. For incorrectly bisected triplets, a 2 × 2 × 2 was realized with the factors distance of the second number to the numerical middle (i.e., far: 2-8, e.g. in 12_13_21, near: 0.5-1.5, e.g. 13_16_21) and bisection possibility (i.e., the existence of an integer in the middle; bisectable: e.g., 12_14_18, non-bisectable: e.g., 12_14_17) being manipulated. The remaining factor, with vs. without articulatory suppression, was consistently applied to both the correctly and incorrectly bisected condition. The experiment was set up in four runs (two with concurrent articulation and two without) with run order varied across participants in a Latin square design.

The 335 triplets of the Nuerk et al. [13] stimulus set exclusively involving two-digit numbers were used in this experiment. These numbers ranged from 12 to 98 with the numerical range of the triplets spanning between 4 and 18. All triplets were presented in Arabic notation. As the exclusion of triplets containing one-digit numbers resulted in a substantial change in item characteristics per experimental condition, a subset of 38 triplets was chosen from each condition for having matched stimulus properties (see Table 1). This resulted in a set of 304 critical triplets of which each one was presented once in each run.

**Table 1:** Stimulus properties (means with standard errors in parentheses) for bisectable triplets used in the analyses

	Bisectable triplets			
	
	Multiplicative		Non-multiplicative	
		
	Small range	Large range	Small range	Large range
Sum	173.61 (11.58)	173.29 (9.44)	173.61 (9.35)	173.29 (8.66)
log sum	2.21 (0.03)	2.21 (0.03)	2.21 (0.03)	2.21 (0.03)
Sum log	5.12 (0.11)	5.18 (0.08)	5.19 (0.08)	5.18 (0.08)
Distance #3-#1	6.11 (0.20)	14.68 (0.34)	6.05 (0.02)	14.42 (0.29)
Distance #2-#1	3.05 (0.10)	7.34 (0.17)	3.03 (0.10)	7.21 (0.14)
Distance #3-#2	3.05 (0.10)	7.34 (0.17)	3.03 (0.10)	7.21 (0.14)
Parity #1	0.08 (0.14)	0.29 (0.12)	0.24 (0.14)	0.26 (0.12)
Parity #2	0.34 (0.12)	0.26 (0.12)	- 0.21 (0.14)	- 0.11 (0.14)
Parity #3	0.08 (0.14)	0.29 (0.12)	0.24 (0.14)	0.26 (0.12)
Mean parity	0.17 (0.10)	0.28 (0.08)	0.09 (0.11)	0.14 (0.10)
Parity homogeneity	- 0.08 (0.14)	0.05 (0.16)	- 0.05 (0.16)	0.33 (0.16)
Decade crossing	0.16 (0.14)	1.00 (0.00)	0.18 (0.14)	1.00 (0.00)
Decade inclusion	- 0.13 (0.14)	0.00 (0.16)	- 0.11 (0.14)	- 0.08 (0.14)

*Sum *indicates the overall sum of the three numbers constituting a triplet; *Log sum *reflects the logarithm of this overall sum; *Sum log *denotes the sum of the logarithms of the individual numbers; *Distance # 3-# 1 *gives the absolute distance between the two outer numbers of the triplet, correspondingly for the other distances; *Parity # 1/2/3 *reflects the "average" parity of the respective numbers (even coded by +1, odd coded -1). *Mean parity *indicates the average parity of the three numbers and *Parity homogeneity *indexes whether all three numbers have the same parity or not (coded +1 vs. -1, respectively); *Decade crossing *indicates whether a triplet crosses a decade boundary (coded +1 for decade crossing, -1 for no decade crossing); *Decade inclusion *denotes whether the triplet involves a multiple of 10 or not (coded +1 vs. -1, respectively).

### Procedure

After having signed an informed consent form, participants were seated approximately 50 cm in front of a 17'' screen with their head on a chin rest. The task instructions were given orally and focused on both speed and accuracy. Participants performed 25 practice triplets which were not part of the critical item set prior to each run. Each run consisted of 19 blocks and each block including 16 triplets. With 2 triplets of each condition per block trial conditions were counterbalanced and triplets were presented in randomized order within each block. The sequence of blocks was altered in each run.

At trial onset, the fixation mark "_ _" was displayed for 500 ms followed by three numbers (e.g. 15_18_21) which remained on the screen until a response occurred. Stimulus size was 1.2° of viewing angle in height and 4.3° in width which corresponds to font size 25 (font: System). Response keys were the upper left "¬/¦" EBCDIC key and the upper right "-" key of the numeric keypad of a standard BS 4822 QWERTY keyboard to be pressed with the thumb of either the right or the left hand. No feedback was given. Participants had to begin uttering "pataka" already three seconds before the next block was started.

## Results

All participants managed to achieve an overall error rate of less than 12% (*M *= 6.8%, *SD *= 3.4%, range 1%-11%) and were included in the analyses. Only latencies followed by a correct response were included in the RT analyses. Trimming first excluded RTs shorter than 200 ms and longer than 6000 ms. In a second step, RTs falling below or above 3 *SD *of a participant's mean were eliminated. Trimming resulted in an additional loss of 4.9% of the RT data.

As the current study aimed at investigating the influence of concurrent articulation on the retrieval of multiplication facts, only data for the correctly bisected triplets were analysed for specific effects of concurrent articulation on the processing of multiplicative triplets. As already outlined above, Cassel and Dallenbach [30] summarized that auditory distraction may result in, either slowing, speeding or no effect at all on overall response latencies. However, we were not concerned with the effect of articulatory suppression on overall performance. Instead, the specific interaction of articulatory suppression with multiplicativity was of interest. Thus, to specifically evaluate this interaction, the analyses were based on RT controlled for the overall effect of articulatory suppression. In order to have an independent estimate of the actual effect of concurrent articulation, the articulatory suppression effect for the *incorrectly *bisected triplets was computed for each participant individually. Please note that this included RT for all triplets followed by a correct no response (e.g., 21_25_27). According to the experimental design this represented one half of the stimulus set. In a next step, the individual estimated effect of articulatory suppression was added to the individual participants' average RT for both multiplicative and non-multiplicative correctly bisected triplets. Thereby, these RTs rates were controlled for the individual overall effect of articulatory suppression, irrespective of comprising either slowing or speeding. Error rates were pre-processed in a similar way. Additionally, error rates were arcsine-transformed prior to the analyses to approximate normal distribution of these scores. Then the resulting RT and ER values were submitted to a 2 × 2 repeated-measures ANOVA with the factors articulatory suppression (with vs. without), and multiplicativity (yes vs. no).

**Figure 1:**
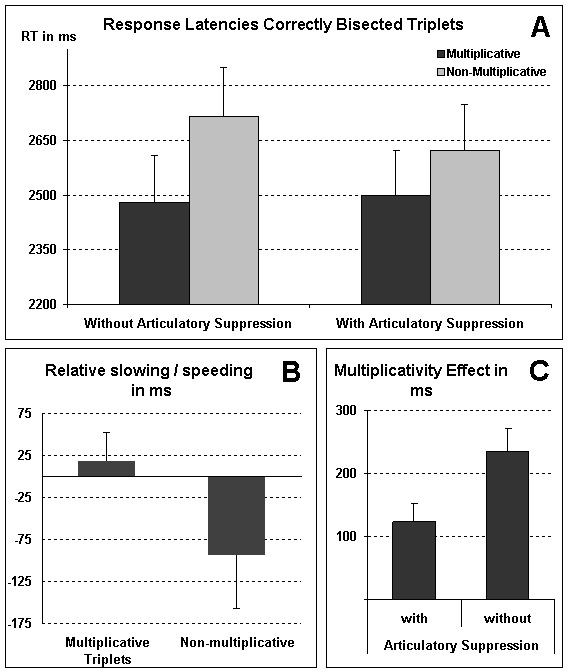
**Results of RT analyses**. Response latencies for correctly bisected triplets separated for the experimental conditions after controlling for the overall effect of concurrent articulation as estimated by the articulatory suppression effect for incorrectly bisected triplets (Panel A). A significant main effect of multiplicativity can be observed in both conditions (with and without articulatory suppression). Panel B depicts the relative slowing of multiplicative resp. the speeding of non-multiplicative triplets when general speeding due to articulatory suppression is partialled out as estimated by the effect of concurrent articulation on RT for incorrectly bisected triplets. It can be observed that articulatory suppression indeed slows down multiplicative triplets specifically. Finally, Panel C illustrates the reduction of the beneficial multiplicativity effect due to concurrent articulation. Error bars indicate 1 Standard Error of the Mean (SEM).

The main effect of multiplicativity was reliable for response latencies only [RT: *F*(1, 11) = 69.63, *p *< .001, *η*_p_^2 ^= .86, errors: *F*(1, 11) = 2.50, *p *= .14, *η*_p_^2 ^= .19, see Figure 1, Panel A]: multiplicatively related triplets were evaluated faster (2490 ms vs. 2669 ms) but not more accurately than unrelated triplets (6.0% vs. 7.7%). Due to control for the overall effect of articulatory suppression on classification performance, the main effect of articulatory suppression was not significant in both the RT analysis [F(1, 11) = 1.02, *p *= .33, *η*_p_^2 ^= .09] and the error analysis [F(1, 11) < 1]. For raw RT and error rates the main effect of articulatory suppression was marginally significant in the RT analysis [F(1, 11) = 3.82, *p *= .08, *η*_p_^2 ^= .26] and reliable in the error analysis [F(1, 11) = 11.86, *p *< .01, *η*_p_^2 ^= .52]: responses became less accurate (8.4% vs. 5.4%) but tended to be faster (2504 ms vs. 2598 ms), while participants performed in the concurrent articulation condition.

In accordance with our expectations, the ANOVA revealed that concurrent articulation indeed exhibited differential influences on multiplicative related and unrelated triplets, as indicated by the significant interaction of multiplicativity and articulatory suppression for response latencies [*F*(1, 11) = 4.87, *p *< .05, *η*_p_^2 ^= .31, errors: *F*(1, 11) < 1, *η*_p_^2 ^= .08]. Concurrent articulation led to a specific relative slowing of multiplicative related triplets (+19 ms) whereas it resulted in a specific relative speeding for non-multiplicative triplets (-93 ms, see Figure 1 Panel B). Neither the slowing for multiplicative nor the speeding up for non-multiplicative triplets was reliably larger than 0 per se [multiplicative: *t*(11) = 0.93, *p *= .37; non-multiplicative: *t*(11) = -1.57, *p *= .14]. However and most importantly, the direct difference between the numerically slowed multiplicative and the numerically speeded non-multiplicative triplets was statistically reliable [*t*(11) = 2.21, *p *< .05]. This indicated a significant specific interaction contrast in the 2 × 2 design with the factors multiplicativity and concurrent articulation for correctly bisected triplets. Importantly, this interaction was *not *driven by general RT differences between runs with and without articulatory suppression as the main effect of articulatory suppression was not significant after controlling for the overall effect of concurrent articulation^3^. Taken together, it is important to note that the interaction of multiplicativity and concurrent articulation was reliable also after controlling for general differences in processing speed as this indicated that the reduction of the multiplicativity effect under articulatory suppression was not an artefact of the generally faster reaction times for this condition. Instead these results corroborate the notion of a specific detrimental influence of concurrent articulation on the processing of arithmetical facts.

## Discussion

The present study was set up to disambiguate the important finding that multiplication is impaired by concurrent articulation [10] and to further specify the origin of this impairment. Therefore, we combined articulatory suppression with the verification version of the number bisection task (NBT). Nuerk and colleagues [13] found multiplication fact knowledge to facilitate performance in the NBT (i.e., the multiplicativity effect: multiplicatively related triplets were classified faster than non-related triplets, see also [14-16]). However, as the NBT does not require intentional access to and active production of multiplication results, no activation of the verbal output lexicon should occur. This makes the results pattern observed in the current study informative as regards the nature of mental multiplication fact representations. We reasoned that an interaction of the multiplicativity benefit and concurrent articulation indicating relative slowing of multiplicatively related triplets would index an at least partially verbal/phonological nature of the storage of multiplication facts in long-term memory.

In accordance with this line of thought, we found that multiplicatively related triplets were indeed responded to specifically slower under articulatory suppression. Yet, this relative slowing under concurrent articulation did not eliminate the multiplicativity benefit. Nevertheless, this seems to support the phonological storage hypothesis of multiplication facts as, for instance, put forward by the Triple Code Model (Dehaene & Cohen [2,3]; see also [10] for experimental data). When the articulatory loop is loaded by articulatory suppression, activation of verbally represented multiplication fact knowledge is impaired so that multiplicative triplets may have to be evaluated via the default strategy of magnitude manipulation as well. However, the relative slowing due to concurrent articulation was smaller than the multiplicativity benefit and thus did not completely balance the multiplicativity benefit. Yet, it is known that impairment due to concurrent articulation depends on the type of concurrent articulation and cannot be assumed to inhibit the processing of auditory-verbal information in the phonological loop completely (cf. [33-35]). Even though we are confident that the specific procedure of concurrent articulation we adopted (i.e., intermittent suppression using changing state syllables) is known to impair phonological processes substantially (cf. [33,36]) the preservation of the multiplicativity effect under concurrent articulation indicated that the loading of the articulatory loop, which is part of the verbal system of number processing (cf. Figure 2, p. 88, [2]) may indeed have been incomplete. Nevertheless, Saito [33] also states that articulatory suppression, such as repeated uttering of non-word syllable strings, is "enough to decrease recall to a certain level" (p. 575). In line with this, the current data substantiate and extend previous results by Lee and Kang [10]. On the one hand, we were able to replicate the concurrent articulation effects reported by Lee and Kang [10] in a multiplication task using a comparable non-word string for suppression as employed by Lee and Kang [10]. On the other hand, the present data are also informative as to differentiate between verbally mediated storage of multiplication facts or verbally mediated access to multiplication fact knowledge. As the current task did not require participants to actively produce any result, the verbal retrieval related output lexicon should not have been activated. Thereby, the observed relative slowing for multiplicative related triplets under articulatory suppression resulting in a reduced multiplicativity effect cannot be attributed to impaired verbal access to participants' multiplication fact knowledge. Instead, these data indicate that multiplication facts are represented at least partially in a verbal format.

An interesting, although not reliable finding was that the effect of articulatory suppression tended to result in shorter response latencies when compared to baseline performance. On the other hand, concurrent articulation led to an increase in error frequency, in particular for bisectable trials. As this pattern of results suggests a speed accuracy trade-off (SATO), additional univariate analyses of covariance (ANCOVA) were conducted to evaluate the relation of RT and error likelihood. In case of a SATO accounting for the reliable main effect of articulatory suppression in the error data, this effect should disappear when incorporating the effect of concurrent articulation on RT (i.e., the RT difference dRT between the conditions with and without articulatory suppression) as a covariate in the analysis. However, the inclusion of dRT did not change the main effect of articulatory suppression substantially [ANOVA: *F*(1, 11) = 11.18, *p *< .01; ANCOVA: *F*(1, 11) = 10.32, *p *< .01] arguing against the presence of a SATO in our data.

Interestingly, in their study examining the involvement of working memory in verification of simple arithmetic products, De Rammelaere and co-workers ([31]; see also [32]) also reported faster responses for some experimental conditions under articulatory suppression (see Table two in [32], p. 271). A possible explanation for this finding may be an attention-related argument. Due to the more challenging task in the concurrent articulation condition, it may have been the case that participants' intrinsic alertness was increased, which in turn led to faster responses (e.g., [37,38]). Alternatively, this may reflect a phenomenon called intersensory facilitation [39]. For example, it is known from transcranial magnetic stimulation studies that the rhythmical click of the coil and the tactile stimulation of the skull lead to faster reaction times even when the pulse is applied over task-irrelevant sites (e.g., [40]). Rhythmically uttering "pataka" could have led to a tendency for comparable and unspecific intersensory facilitation in our experiment. In this regard, also the speeding up of non-multiplicative triplets might be accounted for even though this has to remain speculative. For the special case of non-multiplicative triplets it is conceivable that these triplets may be particularly amenable to intersensory facilitation induced by the rhythmical uttering of "pataka" because this rhythm (comparable to the beat of a metronome) may have corroborated successful regular sequential processing from the first to the second and further to the third number of the triplet. In this vein already Cassel and Dallenbach [30] summarized that auditory distraction may speed up reaction times when the distractor occurs regularly. Importantly, such a successful sequential processing is not present for incorrectly bisected triplets, because here the steps from the first to the second and the second number are irregular in as much as the numerical distance of these steps differs. Finally, also no interference from verbal loading should be present because these numbers were not multiplicatively related.

## Conclusions

By combining concurrent articulation with the verification version of the NBT we narrowed down the origin of detrimental effects of articulatory suppression on multiplication fact retrieval. Importantly, our current primary task did not require participants to actively produce any multiplication result. As a consequence, the verbal retrieval related output lexicon should not have been activated. Therefore, our observation of a relative slowing of the multiplicative triplets by articulatory suppression (which reduced but did not eliminate the initial multiplicativity benefit) implies that multiplication facts are stored and represented at least partially in a verbal code.

## Endnotes

1. Distinguishing between these two versions of the phonological storage hypothesis was not at the core of the current study. Both versions assume some kind of auditory-verbal representation of multiplication facts, which should be impaired by concurrent articulation loading the verbal output lexicon, independent of whether this explicitly involves phonological information or not.

2. Please note that the multiplicativity effect can also not be explained by a higher familiarity of the numbers involved in the multiplicative related triplets. To contrast the frequency of occurrence of the constituting numbers of either multiplicative or non-multiplicative triplets we conducted a google survey on all two-digit numbers from 12 to 98. Thereby, we obtained the frequency of occurrence of each two-digit number used in any one of the triplets. Then, the mean frequency of occurrence for multiplicative and non-multiplicative triplets were computed and directly contrasted. A *t*-test revealed that there was no difference in the frequency of occurrence of the numbers involved in either multiplicative or non-multiplicative triplets [*t*(151) = 1.08, *p *= .28].

3. An additional analysis on z-transformed raw RT (z-transformation carried out individually for each participant and separately for the conditions with and without articulatory suppression to control for general RT differences) also confirmed the interaction of concurrent articulation and multiplicativity in the expected direction [*F*(1, 11) = 5.84, *p *< .05, *η*_p_^2 ^= .35]: the multiplicativity effect was smaller under concurrent articulation than without concurrent articulation (0.12 vs. 0.23 z-scores, respectively). Furthermore, additional analyses indicated that the multiplicativity effect was smaller under articulatory suppression than without [*t*(11) = 2.21, *p *< .05, *d *= .1.02, see Figure 1, Panel C] but remained significantly different from zero even under concurrent articulation [*t*(11) = 4.20, *p *< .01, *d *= .24]. The latter indicated that the relative slowing observed for multiplicative triplets under concurrent articulation was considerably smaller than the multiplicativity benefit.

## Competing interests

The authors declare that they have no competing interests.

## Authors' contributions

KM, KW, MHF, and HC conceived the study. All authors participated in its design. KM performed data collection, processing and statistical analyses. KM and EK drafted the manuscript. All authors contributed to the interpretation of the data. All authors read and approved the final manuscript.
